# Individual user involvement at Healthy Life Centres: a qualitative study exploring the perspective of health professionals

**DOI:** 10.1080/17482631.2018.1492291

**Published:** 2018-07-16

**Authors:** Espen Sagsveen, Marit By Rise, Kjersti Grønning, Ola Bratås

**Affiliations:** a Department of Public Health and Nursing, Faculty of Medicine and Health Sciences, Norwegian University of Science and Technology, Trondheim, Norway; b Department of Mental Health, Faculty of Medicine and Health Sciences, Norwegian University of Science and Technology, Trondheim, Norway

**Keywords:** Service user involvement, primary health care, healthy lifestyle, motivational interviewing, qualitative research

## Abstract

The aim of this study was to explore how professionals experience user involvement at an individual level and how they describe involving users at Healthy Life Centres. Four focus group interviews were conducted with a total of 23 professionals. Data were analysed using systematic text condensation. Four themes were identified: (1) Involving users through motivational interviewing; (2) Building a good and trustful relation; (3) Assessing and adjusting to the user’s needs and life situation; and(4) Strengthening the user’s ownership and participation in the lifestyle change process. Motivational interviewing was described by the professionals as a way to induce and ensure user involvement. However, seeing motivational interviewing and user involvement as the same concept might reduce user involvement from being a goal in itself and evolve into a means of achieving lifestyle changes. The professionals might be facing opposing discourses in their practice and a dilemma of promoting autonomy and involvement and at the same time promoting change in a predefined direction. Greater emphasis should thus be put on systematic reflection among professionals about what user involvement implies in the local Healthy Life Centre context and in each user’s situation.

**Abbreviations:** HLC: Healthy Life Centre; MI: Motivational Interviewing; NCD: Non-communicable diseases; STC: Systematic Text Condensation. SDT: Self-determination theory

## Introduction

One of the major health challenges of the twenty-first century, when it comes to human suffering, mortality and the negative impact on the socio-economic development of countries, is the increasing number of non-communicable diseases (NCDs) (Riley et al., 2016; Sommer et al., 2015; WHO, 2009, 2013, 2014). The rise of cardiovascular diseases, cancers, chronic respiratory diseases and diabetes is primarily caused by lifestyle-related behavioural risk factors: tobacco use, physical inactivity, unhealthy diet and harmful use of alcohol (Ding et al., 2016; Riley et al., 2016; WHO, 2014, 2015). To meet these challenges, national and international authorities have incorporated health-promoting strategies into public policies (Ministry of Health and Care Services, 2013b; OECD, 2015; United Nations, 2015; WHO, 2013). The World Health Organization (WHO) has proposed a number of policy options and actions for implementation by the Member States (WHO, 2013, 2014), stating that the governments are the guardians of a population’s health and thus are responsible for ensuring that institutional, legal, financial and service arrangements are provided for the prevention and control of the NCDs. Examples of national initiatives aiming for the adoption and maintenance of healthy lifestyle behaviours are physical activity programmes such as the “Exercise on Prescription” (Sørensen, Sørensen, Skovgaard, Bredahl, & Puggaard, 2011) and “Physical Activity on Prescription” (Olsson et al., 2015; Rödjer, Jonsdottir, & Börjesson, 2016) programmes initiated in Sweden, Denmark, the Netherlands and Finland. Many countries have also developed policies to promote healthy eating (Capacci et al., 2012).

Service user involvement is considered essential in promoting people’s health and ensuring the quality of health services (Dent & Pahor, 2015; Rise & Steinsbekk, 2016; Snyder & Engström, 2016; Tenbensel, 2010; WHO, 1986; Williamson, 2014), and is described as one of the ideals of contemporary health care (Longtin et al., 2010). A number of studies have shown that user involvement has potential benefits in changing health-related behaviours, empowering citizens to take greater responsibility for their own health, controlling health costs, improving the quality of health care provision and increasing patients’ satisfaction and adherence to treatment (Angel & Frederiksen, 2015; Castro, Van Regenmortel, Vanhaecht, Sermeus, & Van Hecke, 2016; Phillips, Street, & Haesler, 2016; Williamson, 2014). Service user involvement encompasses involvement at an individual level, such as in decision-making regarding individual treatment, or at an organizational level in the development of health care services and policy (Rise & Steinsbekk, 2016; Snyder & Engström, 2016; Tritter, 2009). To ensure the involvement of service users and health professionals’ duty to do so, some Western countries, such as Norway, have legislated user involvement (Health and Care Services Act, 2011; Health Authorities and Health Trusts Act, 2001; Patient- and Users’ Rights Act, 1999). Moreover, the obligation to involve users is advocated in health policy documents, both in Norway and internationally (Ministry of Health and Care Services, 2015b; Mockford, Staniszewska, Griffiths, & Herron-Marx, 2012; NHS England, 2016; NHS England/Public Participation Team, 2015).

In Norway, user involvement constitutes an important part of a health reform (the Coordination Reform) implemented to strengthen health promotion and the prevention of NCDs (Ministry of Health and Care Services, 2009, 2011, 2013a, 2015b; Romøren, Torjesen, & Landmark, 2011). In this reform, Healthy Life Centres (HLCs) have been established in primary health care to promote health and prevent NCDs (Ministry of Health and Care Services, 2011, 2013b, 2015a; Norwegian Directorate of Health, 2016). The HLCs are easily accessible, and people can attend the service through referral from a general practitioner (GP) or other health care providers or by self-referral (Norwegian Directorate of Health, 2016). The HLCs offers knowledge-based support for changing living habits and coping with health challenges through individual- and group-based counselling and activities, and use client-centred counselling approaches to promote the participants’ internal motivation, empowerment and coping (Abildsnes, Meland, Samdal, Stea, & Mildestvedt, 2016; Følling, Solbjør, & Helvik, 2015; Lerdal, Celius, & Pedersen, 2013; Norwegian Directorate of Health, 2016).

The Norwegian Directorate of Health (2016) has recommended that HLCs adopt an approach based on salutogenesis (Antonovsky, 1987, 1996) and use motivational interviewing (MI) as a counselling approach. Research looking at the evidence of behavioural interventions aiming to promote physical activity and healthy eating among overweight and obese adults has found that interventions emphasizing a person-centred and autonomy-supportive communication style through MI and Self-Determination Theory (SDT) are associated with long-term positive effects (Grieco, Sheats, Winter, & King, 2014; Samdal, Eide, Barth, Williams & Meland, 2017). Beyond the legally required duty to involve service users, the HLC guidelines give recommendations on how users should be involved in the planning, delivery and evaluation of services (Health and Care Services Act, 2011; Patient- and Users’ Rights Act, 1999; Norwegian Directorate of Health, 2016).

A number of studies have investigated how health professionals experience user involvement at an individual level and how they work to enhance involvement, such as within mental health, cancer, diabetes and coronary care (Millar, Chambers, & Giles, 2016; Snyder & Engström, 2016). Findings have shown that the main motive for health professionals to initiate user involvement is to gain access to user knowledge as an alternative to professional knowledge or as support for professional knowledge (Sahlsten, Larsson, Sjöström, & Plos, 2009; Solbjør & Steinsbekk, 2011). However, the responsibility to deliver evidence-based or high-quality care, together with respecting service users’ right to make decisions, is sometimes described as conflicting (Shortus, Kemp, McKenzie, & Harris, 2013; Solbjør & Steinsbekk, 2011). Ensuring high-quality care is described as an argument for limiting user involvement and for professionals to decide and to exert control over patient care (Larsson, Liljedahl, & Gard, 2010; Shortus et al., 2013; Solbjør & Steinsbekk, 2011; Tobiano, Bucknall, Marshall, Guinane, & Chaboyer, 2015; Tobiano, Marshall, Bucknall, & Chaboyer, 2016), valuing professional knowledge above user knowledge (Solbjør & Steinsbekk, 2011). Although actively involved patients were considered valuable to enrich the professionals’ work, it is also looked upon as time-consuming and increasing the workload (Arnetz, Winblad, Arnetz, & Höglund, 2008; Arnetz & Zhdanova, 2015; Solbjør & Steinsbekk, 2011).

Different reasons are given for involving service users in health services, and individual user involvement in treatment decisions is described as one category (Tritter, 2009). To differentiate the aim of involvement, Tritter provides a framework in relation to the dimensions: direct—indirect; individual—collective; proactive—reactive (Tritter, 2009). By linking positive and negative descriptions from service users about how they would like to be involved, Thompson developed a taxonomy with five discrete levels of patient-determined involvement (Thompson, 2007). These levels of involvement are labelled “Non-involvement”, “Information-seeking/receptive”, “Information-giving/dialogue”, “Shared decision-making” and “Autonomous decision-making” (Thompson, 2007). Thompson compared this taxonomy with existing theories of patient involvement, resulting in five parallel levels of professional-determined involvement, labelled “Exclusion”, “Information-giving”, “Consultation”, “Professional-as-agent” and “Informed decision-making”. Further, the patient’s desire for involvement can be effected only through a matching willingness by professionals, labelled as co-determined involvement (Thompson, 2007).

In an HLC setting, however, little is known about how professionals perceive and work with user involvement at an individual level to promote lifestyle changes. Since user involvement is highly dependent on the professionals’ knowledge and attitudes towards involving users in their daily work (Longtin et al., 2010; Phillips et al., 2015; Rise & Steinsbekk, 2016; Wiig et al., 2013), the professionals’ perspective is important. Hence, the aim of this study was to explore how HLC professionals experience user involvement at an individual level and how they describe involving the service users in individual- and group-based counselling and activities at HLCs.

## Methods

In this qualitative study, we used semi-structured focus group interviews to explore professionals’ perspectives with involving service users at Norwegian HLCs. Focus group interviews facilitate interaction between the participants and help to initiate recall, which is useful when doing an explorative study (Krueger & Casey, 2014). The study was conducted in Norway from September 2015 to May 2016.

### Setting and sampling

The sampling was strategic, recruiting participants from HLCs in both rural and urban municipalities in Central and South-Eastern Norway. The sample of HLCs should include both well-established and new centres, as well as centres differing in size (regarding numbers of employees and inhabitants the HLC served). Only participants from HLCs offering a 12-week follow-up period with individual health counselling were eligible. An additional inclusion criterion was that participants had experiences with individual- and group-based counselling and activities related to physical training and dietary behaviour or tobacco cessation. Four focus group interviews were conducted. Participants in focus group interviews one and two were recruited in collaboration with two local health coordinators, who informed the participants about the study and then sent the names of the participants who volunteered to participate to the first author (ES). Participants to focus groups three and four were recruited directly by the first author by telephone. The contact information of the participants was found on the websites of the Norwegian Directorate of Health and the municipalities in which the participants were employed. Those who agreed to participate received an email with an information letter, and all signed a written consent form before taking part in interviews.

### Data collection

The interviews included focus groups with 4 to 8 participants, each lasting for 90 minutes. The interviews took place at different locations. Focus group one was conducted in the location of one of the HLCs. Focus group interviews two, three and four took place at meeting rooms located at a university campus and at a public community and conference centre. The groups were organized in line with the inclusion criteria, with a mix of new and well-established HLCs and participants of different ages and experiences. Practical considerations were also taken into account, such as group size, travel distance and finding meeting dates, times and places that were convenient for the participants.

All interviews were conducted according to an interview guide developed by the first author, including a literature review and discussions with co-authors, one of them with extensive research experience on user involvement (the second author, MBR). The interviews were initiated using open-ended questions inviting the participants to tell about their own experiences, and participants were encouraged to discuss and illustrate their experiences with user involvement with examples from their practice. See Table I for examples of topics from the interview guide. These questions were used only as a guide, and the sequence was dependent on the participants’ response to previous questions.

**Table I.** Main topics in the interview guide.

(1) What do you understand by user involvement at the HLC?
(2) What kind of experiences do you have with involving the users?
- examples of positive and/or negative? - examples of challenges (if so, how)? - can user involvement be in conflict with your professional judgement (if so, how)?
()(3) What kind of meaning or significance do you think user involvement has?
- benefit or effect? - influence on the service or on the way you work? - can user involvement have an intrinsic value (if so, how)? - can user involvement be health promoting (if so, how)?
()(4) What is needed of you to facilitate for user involvement?
- to what extent do the users want to be involved (why or why not)? - what are the prerequisites for user involvement?

The participants took an active part in the discussions by sharing personal experiences and reflections. Some knew each other, but only a few worked together on a daily basis. The participants’ familiarity with each other may have made them feel comfortable and helped them open up for the sharing of personal experiences. On the other hand, the participants’ experience of knowing each other and working together may also have contributed to homogeneity in experiences and opinions. The discussions did not reveal any clear disagreements about the topic, but the participants had different experiences with user involvement due to organizational structures such as number of service users and the organization of HLC activities.

The first author operated as the moderator, ensuring that everyone had the chance to express his or her view. The fourth author (OB) acted as an assistant moderator, making notes throughout the group discussions and asking supplemental questions at the end of the interviews. All interviews were audio-recorded and transcribed verbatim. The study was approved by the Norwegian Data Protection Official for Research (Project no. 43,803).

### Data analysis

After each interview, the first author listened to the digital recording and wrote a summary. After four interviews, it was concluded that no new or relevant data seemed to emerge, and the information gathered was found to be sufficiently saturated for analysis (Malterud, 2012).

Analysis was conducted as collaborative negotiations between the four authors. Systematic Text Condensation (STC) inspired by Giorgi’s approach as described by Malterud (2012) was used. STC was chosen because it offers a process of intersubjectivity, reflexivity and feasibility during the analysing of data, and it is a structured and well-described systematic method for analysing qualitative data. Further, STC focuses on the thematic analysis of meaning and content of data across cases, and thereby is useful for our study. The STC procedure consists of four steps (Malterud, 2012). First, all transcripts were read by all four authors to establish an overview and to gain a general impression of the data, searching for preliminary themes related to the professionals’ involvement of users. At this stage, it was important that the researchers tried to bracket their preconceptions and meet the data with an open mind, demonstrating awareness to the participants’ voices. After reading the transcripts, all authors met to discuss the preliminary themes found. Examples of preliminary themes were: “Professionalism”, “Ownership” and “The Meeting”. Next, the transcripts were reviewed by the first author to identify meaning units representing different aspects of the participants’ experience with involving users. Third, the first author classified and sorted the meaning units into code groups, followed by a common agreement between the authors about the content of the codes. Further, the first author systematically sorted the meaning units of the actual code groups into a few subgroups. Then, the content of every subgroup was reduced into a condensate—an artificial quotation maintaining as far as possible the original terminology used by the participants. Examples of code groups and subgroups were as follows: (1) the code group “Professional assessment and knowledge” with the subgroups “Individualization” and “Balance between what the users want and the professionals’ knowledge”, and (2) the code group “Ownership to own change process” with the subgroups “Responsibility” and “Preparing the ground for user involvement”. After finishing the condensation, illustrative quotations were identified. Finally, the condensed contents were synthesized to generate generalized descriptions and concepts (recontextualized) concerning professionals’ experiences with involving users at the HLCs, described as the final themes in the presentation of results. The interpretations and findings were validated by the research group against the initial transcripts to ensure that the synthesized result still reflected the original context. See Table II for an example of the analysis process.

**Table II.** Example of stepwise analysis using STC.

Step 1: Preliminary theme	Step 2: Identifying and sorting meaning units	Step 3: Condensation	Step 4: Final themes
Professionalism	“When I think about user involvement during the process, then I feel it is present all the time, because we try to be where the user are and adjust to the user in front of us.” “I experience that the clear majority understand that you cannot adjust everything to all participants. You have a sort of minimum common plan, and that is what they have decided to join.” “The goal is, as I see it, to tailor a plan for every single user.”	You have to keep yourself updated and as professionals we know what is effective training and healthy diet, and we have a responsibility to offer a service that is evidence-based. So, you have to balance between what the users want and our professional knowledge, if this is in conflict. I think, however, user involvement is present, because our goal is to tailor and individualize the service to every user’s needs.	Assessing and adjusting to the user’s needs and life situation.

During the analysis process, preliminary results were presented and discussed with a research group focused on patient education and involvement. Preliminary results were also presented and discussed at a national HLC conference with a focus on user engagement and at a national seminar with user representatives.

Quotes from the transcripts were translated into English by the first author (ES) and then double-checked by the other authors to verify the meaning content. Quotes are used in the result presentation to elaborate and illustrate the findings. Because of only one male being represented in the sample, gender is not attached to the citations to anonymize the data.

## Results

Twenty-three professionals from 23 HLCs from both rural and urban municipalities in Central and South-Eastern Norway participated in a total of 4 focus group interviews. Details of the sample are listed in Tables III and IV. When asked what they understood by user involvement and how they involved users in the Healthy Life Centres (HLC), the professionals described four main themes: (1) Involving users through motivational interviewing; (2) Building a good and trustful relation with the user; (3) Assessing and adjusting to the user’s needs and life situation; and (4) Strengthening the user’s ownership and participation in the lifestyle change process. These four themes will be elaborated below. Quotes from the data material are presented to illustrate the findings.

**Table III.** Demographic characteristics of the participants (N = 23).

Characteristics	Number of informants
**Gender**	
Male	1
Female	22
**Age**	
18–29	6
30–39	5
40–49	7
50–59	3
> 60	2
**Profession**	
Physiotherapist	11
Nurse	6
Educationalist	2
Clinical dietitian	2
Occupational therapist	1
Bachelor’s degree public health	1
**Years of Seniority at the HLC**	
< 2	7
2–4	7
> 5	6
**Percentage of full-time equivalent**	
20–30	6
40–50	7
60–70	1
90–100	9

**Table IV.** Characteristics of the HLCs (N = 23).

Characteristics	Number of HLCs
**Established**	
2003 or before	2
2004–2006	3
2007–2009	3
2010–2012	9
2013–2014	6
**Number of positions**	
1	9
2	6
3	5
4	1
9	1
10	1
**Number of inhabitants served**	
1350–4999	7
5000–9999	7
10,000–19,999	4
20,000–49,999	3
50,000–200,000	2

### Involving users through motivational interviewing

When the participants described how they involve the users, they linked this to the health consultation and their use of motivational interviewing (MI) as a conversation technique and method. The participants described MI as a way to induce and ensure user involvement. As they saw it, the goal for the health consultation and the use of MI was that the users should be the ones who control the direction of the conversation and the ones to come up with the proposals for change. The participants saw it as their main goal at the HLC to help people make other or new choices to achieve change. One physiotherapist said:

“I think the conversation technique we use with MI certainly encourages user involvement, because it is based on the user’s preferences. It is the users themselves who shall make the proposals of change.”

The participants described MI as an important tool in the health consultations to find the users’ needs and wishes. The goal, as they said, was to use MI to guide the users to be able to see for themselves what they need. At the same time, as they talked about the user being the one controlling the direction of the conversation, they stated that they also had a responsibility to keep the conversation on track and have a sort of control over it. As part of this, the participants talked about user involvement as a basis for the HLC, since, as they saw it, everything they did at the HLC should be based on what the user wants, as described by one of the physiotherapists:

I think that user involvement is maybe the foundation for the HLC. That everything you do should be grounded on what the user wants, since you have MI in the bottom of the conversation and that you make a plan grounded on this conversation and what kind of problem the user has. In this way, I feel maybe that user involvement is in the centre of the whole service.

Although the participants described MI as an important tool and method to ensure user involvement, they also said that MI was not “the solution of everything”. To know how to practise MI, which they characterized as a complex skill, in the right way was described as important. MI was further said to be something learned with considerable practice over time. However, the participants also saw it as essential to focus on how they actually met the users, as noted by one experienced educationalist:

“It is a bit worrying if MI becomes like ʻif you have that, then you have rescued everythingʼ. But it is a very good tool to start with to get to know each other.”

### Building a good and trustful relation with the user

According to the participants, building a good and trustful relation was an essential part of involving the users. The participants described the first meeting in the health consultation as especially important, since their experience was that the first impression was crucial for whether the users wanted to continue the contact with the HLC. As part of the first meeting, the participants described that they “explored the user’s perspectives” and “showed curiosity and genuine interest” using MI. As a way of showing curiosity and interest, they talked about being present in the situation by actively listening and spending enough time at the health consultation. One nurse with long-time experience said:

The first I thought of when you said user involvement, I was directly into the practical work and how we actually meet them at the HLC. That we in our daily work are especially aware of the user being in the centre of our attention. So I was directly into the health consultation and that you all the time make sure that we actually work like we have set ourselves as goal to work, to be explorative towards the individual.

Further, they emphasized the importance of involving the users as equal partners. The participants described that they wanted the users to feel that they were seen, listened to, respected and met without prejudice, irrespective of background and reasons for attending the HLC, as expressed by one physiotherapist:

The HLC is based a lot on user involvement. That you ask and that you are totally dependent on that you see the other person who stands in front of you as an equal individual … and that you actually get used to listening to the user, as the user has been used to listening to the professionals as an authority before. You are much more equal.

To make their knowledge as professionals and the service they offer at the HLC trustworthy, the participants said it was important to gain the users’ confidence. Some participants described that to gain confidence, it was crucial to be open to and consider other problems than the ones just directly related to the users’ need for lifestyle change. The participants exemplified this by describing that many users told about mental challenges in their lives. The participants talked about user involvement as having the courage to listen, before they referred the user to other services, to prevent the perception that the users were sent from one service to another. One physiotherapist described it this way:

It is not often that you have a new user that expresses: “I need help with my exercise and that is the only problem in my life.” It is a result of many factors that we should have the courage to feel about and take into consideration. Of course we need to know where our limits are, but we are always allowed to listen, at the same time as we should be careful to not give to much advice related to other professions’.

However, some participants said that the building of a good and trustful relation was disturbed by the use of standardized forms, especially during the health consultation. The use of forms was described as disturbing on the communication process since it made it more difficult to individualize the conversation and to gain the users’ confidence. Some participants said, however, that they never used the forms, due to language and cultural challenges. One of the participants, a physiotherapist, exemplified the dilemma between the use of standardized forms and the wish to meet the user without any predefined questions by saying:

It is, I think, a tension between that we have to do surveys of what we do as part of research, because we need statistics, we have to be able to evaluate the service … and because of this we maybe need to use questionnaires, and between just meeting the person and starting from where the person is.

### Assessing and adjusting to the user’s needs and life situation

The participants emphasized the importance of tailoring and individualizing the service for every single user. They further described it as a main task for the HLC to build upon the users’ interests, such as by guiding them into activities outside the HLC. This was seen as a part of user involvement at the HLC, as expressed by one experienced educationalist working in a small municipality:

The goal is, as I see it, to tailor a plan for every single user. And to be able to do this, you need to know what already exists in the municipality. It doesn’t need to be the HLC that offers the plan. Many volunteer organizations run low-threshold activities. Or the users can come with a tip for new groups or activities, which can be started because it is absent in the municipality. So in this way to tailor around the user, it is a good example [of] how it is user involvement from the start, which makes it easier to motivate them for further action.

A challenge in individualizing the service, they said, was that many of the HLCs did not have so many activities to offer. Focusing on user involvement by promoting the users’ own needs and wishes could therefore lead to expectations they were not able to fulfil, as described by one nurse from a small HLC and municipality:

I think we cannot fulfil all the wishes. When the users take an active part, it creates some expectations too. We shall adjust it for them, but inside our framework. So many users have high expectations about courses or swimming pools or many other activities, and then the HLCs maybe have only a few provisions because they are small.

When asked about how they involved users in the planning of physical exercise groups and diet-courses, the common answer was that they involved users in this to a small extent, and that the HLC professionals said they already had a plan prior to the training and the courses. One reason for not involving the users, they said, was that they thought they met the needs of the target group. The participants also described that their impression was that the users understood that they could not customize the activity to everyone’s needs, as described by one of the physiotherapists:

My experience is that the clear majority understand that you cannot adjust everything to all participants. You have a sort of minimum common plan, and that is what they have decided to join. But if you feel the freedom and confidence to speak out and to be seen, and that we adjust on the way, then I feel it is good user involvement, maybe.

Another aspect the participants described as a challenge or obstacle for user involvement was that they were supposed to cover and meet a wide range of needs, conditions and expectations from a large group of users. As examples of different conditions, the participants mentioned the users’ age, physical shape, mental condition, knowledge, cultural background and motivation. The participants said that opening up for “too much” user involvement could lead to feedback that was too specified and related only to the needs of a small number of users, as expressed by one physiotherapist:

But, it is like if everyone shall have their needs fulfilled, then it can be difficult to carry out in practice, so we have to balance between managing and freeing limits … we have a plan and a structure and we have predefined what we think is the best service provision, from a professional point of view. And then we shall integrate this to everyone’s needs and experiences and continuous evaluations.

The participants described their professional knowledge and responsibility as arguments against letting the users decide what to do. To reach the goal of changing the user’s lifestyle or living habits, the participants said that they had the knowledge about what was effective and what would lead to results, as described by a physiotherapist:

And we have, as professionals, if we want to give the ones who come to us an effect of training, then we cannot let the participants in the group walk slowly talking together, if that is what they want, a responsibility to offer training that makes them out of breath and thereby stronger to move on. So, in that we have some responsibility.

And as another physiotherapist said:

“For sure we have user involvement, but we guide them too, because we do know what it requires. But that they realize things themselves can be a good ulterior motive.”

### Strengthening the user’s ownership and participation in the lifestyle change process

The fourth main theme was to develop ownership and participation in the process of lifestyle changes. The participants talked about involving the users to promote their ownership of and responsibility for their own plan, goals and life. To be involved and to feel ownership was also described as a way of making the users feel more committed to follow up their process of health behaviour change. They also thought that involving the users would make them more motivated and increase the users’ chance of succeeding with their own process, as described by one physiotherapist:

It is about ownership of their own plan. Ownership of their life. They know that they should stop smoking, should eat healthy, should exercise, but why shall they do it? What is in it for them—enjoying themselves a lot lying on the couch? So ownership of the plan is for sure important.

The participants said that they expected the users to take responsibility for telling them about what they needed and wanted from the HLC. They also said that they had expectations about the users following the plan and that the users told them if they needed adjustments, such as during the training session. The participants described, however, that some users either did not want to be involved or to express what they wanted, or did not have any thoughts about what they wanted:

It is those persons that want, “Tell me how I should eat, tell me what I should do.” That in a way set him- or herself outside themselves and they do not want to take responsibility. Involvement here can be challenging.

The participants related in their descriptions the users’ will to be involved, from whom the users were referred and how involved the users had been in the referral process. They described the users who referred themselves as the ones most motivated and easiest to involve. The participants further described that some users did not expect to be involved and got confused and sometimes irritated when they were asked about their own thoughts. The participants said that they had experienced that some users expected to be told what to do, and that user involvement for some users could be felt as “scary” and could turn out wrong, when the users did not know what they needed or wanted, as expressed by a physiotherapist:

For many users, I think, if it is too much involvement, it can become a bit “scary” too. One maybe wants an arrangement and plan that are made based on professional knowledge, like “This I can safely apply. I do not know what I need or should manage, but you maybe know that and can tell me that.”

In addition, they described that, for some users, maybe the right thing to do was not to involve them but to tell them what to do and to show them the way. The participants related in their descriptions that this applied to which stage in their process of lifestyle change the users were, as exemplified by the same physiotherapist as above:

Many of the users we meet, maybe what they need is to be held by the hand and shown the way. And if you then, in a way, are given the responsibility to say what you need, it is maybe exactly what is difficult at that stage.

## Discussion

The aim of this study was to explore how HLC professionals experienced service user involvement at an individual level and how they describe involving the service users in individual- and group-based counselling and activities at HLCs. When asked what they understood by user involvement and how they involved users in the HLCs, the professionals described four main themes: (1) Involving users through motivational interviewing; (2) Building a good and trustful relation with the user; (3) Assessing and adjusting to the user’s needs and life situation; and (4) Strengthening the user’s ownership and participation in the lifestyle change process.

### Respect, trust and working in partnership

Our findings showed that trust, respect and working in partnership with adequate time to build relationships are essential to user involvement. This is in line with many other studies showing that an optimal level of involvement depends on both users and professionals having adequate time to build relationships and share knowledge (Angel & Frederiksen, 2015; Leske, Strodl, & Hou, 2012; Rise et al., 2013; Sahlsten et al., 2009; Tobiano et al., 2015). On the other hand, the users’ often complex life challenges and conditions were described as a potential hindrance for user involvement, which is in accordance with other studies pointing out that users’ physical and mental capabilities, severe illness and poor health can impede involvement (Angel & Frederiksen, 2015; Longtin et al., 2010; Rise et al., 2013; Solbjør, Rise, Westerlund, & Steinsbekk, 2013; Tobiano et al., 2015) and health behaviour change (Abildsnes et al., 2017, 2016; Følling et al., 2015). In addition, our findings showed that the building of a trustful relationship and individualization of the service was disturbed by the use of standardized forms which interrupted the communication process.

These results highlight that the relationship between HLC users and professionals might be a facilitator for user involvement when the users’ individual health condition and health challenges are given attention. This finding is underpinned by other studies showing that the extent to which user involvement is desired by the users depends on collaboration, sharing power and the quality of the users’ relationship with the professionals (Leske et al., 2012; Thompson, 2007). This implies that emphasizing competence among HLC professionals in participative communication, which supports relationship building and activation, becomes important to promote user involvement. Further, it highlights the importance of being aware that developing trust, capacity to participate, and consensus around the agenda and goals depend on sufficient time and expertise (Tritter, 2009), and that communicative behaviour and relation-building may be disturbed by protocols and guidelines (Snyder & Engström, 2016).

### Involvement through the practice of MI

A main finding in this study is that the professionals described motivational interviewing (MI) as a way to induce and ensure user involvement. Thus, the professionals are following the recommendations given by the Norwegian Health Directorate about adopting MI as a counselling approach at HLCs (Norwegian Directorate of Health, 2016). MI is described as a collaborative, goal-oriented and person-centred conversation and counselling style for strengthening a person’s own motivation and commitment to change (Miller & Rollnick, 2013). A crucial component of the efficacy of MI is the underlying spirit of MI, described as the set of heart and mind and underlying perspective within which the professionals practise MI (Miller & Rollnick, 2013; Miller & Rose, 2009). The four interrelated elements of the spirit are partnership, acceptance, compassion and evocation (Miller & Rollnick, 2013; Miller & Rose, 2009), which are concepts much in line with user involvement. Both user involvement and MI emphasize collaboration and working in partnership (Miller & Rollnick, 2013; Rise et al., 2013; Snyder & Engström, 2016). Achieving partnership requires activation of both the health care provider and the service user, and the user’s view as an expert must be considered important (Castro et al., 2016; Larsson et al., 2010). Further, a dialogue consisting of a bilateral exchange of experiences and knowledge between service users and health care providers should take place, both in user involvement and in MI (Miller & Rollnick, 2013; Rise et al., 2013; Snyder & Engström, 2016). In addition, the partnership should entail mutual trust and respect (Angel & Frederiksen, 2015; Castro et al., 2016; Rise et al., 2013; Snyder & Engström, 2016).

On the other hand, there are some differences between MI and user involvement, regarding the rationale or arguments behind them. MI is described as a clinical method and goal-oriented guiding style for enhancing intrinsic motivation to move from ambivalence to enduring change (Miller & Rollnick, 2009, 2013; Rollnick, Miller, & Butler, 2008). This means that while MI can be considered as a client-centred counselling method, being goal-oriented in having intentional direction towards change (Miller & Rollnick, 2009, 2013), user involvement can be viewed as an approach, policy or ideology where the process of involvement in itself is valuable (Beresford, 2012; Tritter & McCallum, 2006). User involvement is also a requirement and democratic right in many countries and some therefore argue that involvement is always of value, in its own right, irrespective of its impact (Beresford, 2012; Snyder & Engström, 2016; Staley, 2015).

Hence, when seeing MI and user involvement as the same concept, one may fail to recognize that, for some users, involvement itself may be a goal and thus forget to consider the process as well as an outcome (Angel & Frederiksen, 2015; Tritter & McCallum, 2006). This highlights the need for clarification between the HLC professional and user about the user’s expectations and preferences for involvement, and to what extent involvement is desirable and achievable in each individual case (Angel & Frederiksen, 2015). To what extent user involvement is desired is found to depend on the context; seriousness of health problems; personal characteristics, such as low health literacy and lack of confidence in one’s own capacities; and users’ relationships with the professionals (Longtin et al., 2010; Thompson, 2007). Authors argue that finding the user’s preferences demands high levels of sensibility and flexibility from the health professional in establishing a shared understanding and accommodating the patient’s perspective Collins, Britten, Ruusuvuori, & Thompson, 2007). Further research should therefore investigate the users’ perspectives of involvement in the HLC.

In the present study, the professionals’ practice and understanding of user involvement were described as building relationships, seeing the users as equal partners, emphasizing users’ own behavioural choice and creating ownership. This is in line with the concepts describing both the spirit and content of MI, such as collaboration and honouring autonomy, respect and user involvement (Castro et al., 2016; Miller & Rollnick, 2013; Snyder & Engström, 2016). Our study thus showed that the professionals strongly emphasized using MI to promote health behavioural change (Norwegian Directorate of Health, 2016). On the other hand, when talking about what they understood by the concept of user involvement, their descriptions were less clear. This finding is in line with authors arguing that a fundamental problem affecting user involvement and participation is that the terms tend to be poorly defined and carelessly used, both as theoretical concepts and in practice (Beresford, 2012; Collins, Britten, Ruusuvuori, & Thompson, 2007). It is further argued that user involvement is connected with the disciplines and discourses of politics and political philosophy, of democracy and power, and of citizenship rights and responsibilities (Beresford, 2012). However, it is also found that understandings of involvement often tend to be abstracted from these matters and treated in isolation as technical rather than ideological matters, forgetting that user involvement is not value-free and neutral (Barnes & Cotterell, 2012; Beresford, 2012; Butler & Greenhalgh, 2011). Personal change requires the individual’s active participation in the change process, and the professionals’ use of MI at HLCs contributes to and facilitates for involvement (Miller & Rollnick, 2013). However, a consequence of considering MI and user involvement as the same concept may imply missing the historical, political, ideological and cultural context behind user involvement and how these affect the practice of user involvement (Beresford, 2012).

This is in line with other findings suggesting that user involvement should be viewed not only as isolated activities, but also as a result of reflecting on one’s own view on involving, educating and preparing users, staff and systems (Snyder & Engström, 2016; Tobiano, Marshall, Bucknall, & Chaboyer, 2015). In a primary health care and HLC context, this could imply training staff to support user involvement and to induce explicit discussions among health professionals and leaders about the content of user involvement and how to implement it.

### Professionals’ knowledge and role versus involvement of the user

The findings showed that the professionals try to assess and adjust the service to every user’s needs, promoting the individual as free, active and reflexive. However, they also described how user involvement sometimes conflicts with their professional knowledge and responsibility to help the users change living habits by offering an evidence-based service. These findings indicate that the professionals are facing mutually contrasting and opposing discourses in their practice (Knutsen & Foss, 2011). The professionals seem to be facing a dilemma of promoting autonomy and involvement and at the same time promoting change in a predefined direction. This finding also suggests, as stated by others, that the professionals have to be aware of and adapt to the needs, knowledge and values of the individual user, and to balance the autonomy of the user with an evidence-based practice (Larsson et al., 2010; Taylor, 2009). Our findings showed that the professionals saw the users’ wide range of needs, conditions and expectations as an obstacle for involvement. It was also an argument for not particularly involving the users in the planning of activities.

The participants’ arguments are in line with findings stating that user control can lead to service provisions that meet the needs of some people more than others. Following the opinion of the majority may lead to a service that disadvantages others (Tritter, 2009). In terms of Tritter’s model, the HLC professionals practise indirect and reactive involvement, which entails the gathering of information from service users responding to a pre-existing agenda, where the professionals make the final decisions (Tritter, 2009).

Other studies have also found that it may be difficult to both respect a user’s autonomy and deliver high-quality services, both described as important by the professionals in the present study. To manage both aspects requires a careful balance (Shortus et al., 2013).

These findings also highlight that there may be some challenges due to the nature of the relationship between laypersons and professionals and the difference in situation, power and knowledge (Angel & Frederiksen, 2015). These challenges may arise as a result of the user being the one having the problem and expecting the professionals to be the experts and therefore responsible for solving the problem (Angel & Frederiksen, 2015; Longtin et al., 2010; Thompson, 2007). In line with previous studies, the professionals gave descriptions of how they balance between being paternalistic and promoting the users’ free will (Longtin et al., 2010). The findings showed how the professionals sometimes concluded that the right thing to do, from a professional point of view, was to make decisions for the user.

This finding resonates with Thompson’s taxonomy, describing five discrete levels of patient-desired involvement in consultations, divided into patient- and professional-determined involvement, and spanning from non-involved and exclusion to autonomous and informed decision-making (Thompson, 2007). Our findings showed that the users are given an active role in the health conversations in terms of a professional-determined involvement, where the professionals try to position the users at the level of informed decision-making (Thompson, 2007). This implies that the HLC professionals in our study seem to use their MI expertise as means of helping the users decide for themselves, described by Tritter as direct individual and proactive involvement where the user actively helps to shape his or her own service (Tritter, 2009). Similar to our findings, other studies state that the same user may wish to be involved at different levels and that this may change over time in the same context (Rise, Westerlund, Bjørgen, & Steinsbekk, 2014; Thompson, 2007; Tritter & McCallum, 2006).

### Involvement as personal responsibility

The results showed that the professionals aimed to strengthen the user’s ownership to and participation in the lifestyle change. The professionals described that during the individual health counselling, they laid the groundwork for the users to decide what to do and what should be the goal. According to the professionals, the health consultations are therefore an important means of involving the users and individualizing the experience of their health problems. This is supported by researchers, arguing that as health and medical care become more complex, uncertain and sometimes contradictory, the health consultation aiming to examine the user’s experience becomes increasingly important (Taylor, 2009). The way the professionals involved the users in the health consultations resembles the Self-Determination Theory (SDT) in supporting the individual’s experience of autonomy, competence and relatedness, elements that are considered to promote volitional and high-quality forms of motivation and engagement for activities (Ryan & Deci, 2000, 2017). In line with the SDT, our findings showed that the professionals experienced that the service users’ motivation varied. The professionals’ experience was that users who were self-referred were the ones easiest to motivate. This may resonate with the self-referred user’s motivation being volitional, reflecting their interest or values, whereas the users who were hard to motivate had external motivation, being coerced or pressured into participating at the HLC (Ryan & Deci, 2017).

Thus, supporting the service user towards self-determination means encouraging the user to influence his or her life condition (Angel & Frederiksen, 2015). However, emphasizing an individual focus of involvement and personal responsibility is described as a balancing act between responsibility and blame, which may result in stigmatizing instead of health promotion (Abildsnes et al., 2016; Malterud & Tonstad, 2009; Malterud & Ulriksen, 2011). Thus, acknowledging that some users do not want to be involved can also be regarded as self-determination. This highlights the need to adjust the involvement to the users’ circumstances (Angel & Frederiksen, 2015). Our findings showed that the recommended case-to-case approach and individual adjustment of involvement to optimize user involvement (Angel & Frederiksen, 2015; Rise et al., 2014; Snyder & Engström, 2016) is challenged by organizational issues such as group size and scarce resources, as well as a potential mismatch between the user’s expectations and what the HLC can offer. More research should look into how individual adjustments and user involvement are negotiated against resource use and standardization of services, keeping in mind that user involvement also is political in nature and takes place in a political context (Beresford, 2012).

### Strengths and limitations

This is the first study exploring HLC professionals’ perspectives on user involvement. The participants varied in age, working experience and occupational background, representing both well- and newly established HLCs from urban as well as rural municipalities. These variations strengthen this study. The composition of the focus groups varied as well, with participants in two groups knowing each other, and two other groups in which the professionals were less known to each other. The participants’ familiarity with each other might have contributed to a more relaxed and free-speaking session. On the other hand, former disagreements could have limited the discussion. The level of discussions seemed, however, not to be affected negatively by the group composition. Another strength is that the analysis and writing of the paper were conducted by a group of different researchers. This helped ensure the reliability of the findings.

A limitation is that only one male HLC professional participated in the study. While there are no official statistics describing the gender distribution among HLC staff, a national summary of contact persons in HLCs shows that approximately 10% are male, reflecting the sample in this study. Another possible limitation was the absence of user or public involvement in the research process, such as analysing of data. Including practice-based knowledge through user or public involvement could have enhanced the quality and appropriateness of the research, offering another perspective.

## Conclusion and implication for clinical practice

A trustful relationship was seen as a prerequisite for successful involvement and was highlighted as important, due to the service users’ life situation and conditions. This underpins the importance of participative communication skills among health professionals to promote involvement. The findings showed that MI was described by the professionals as a way to induce and ensure user involvement in the health conversations. However, in seeing MI and user involvement as the same concept, one may fail to recognize that, for some users, involvement in itself may be a goal and not merely a means to achieving lifestyle changes. The results also suggest that promoting the users’ autonomy and user involvement sometimes conflicts with the professionals’ knowledge and their responsibly to help the users change their living habits through offering evidence-based services. Both findings highlight the need for clarification between service user and professionals as to what extent involvement is desirable and achievable. Finally, the results showed that the professionals promoted the users’ ownership and personal responsibility in line with the Self-Determination Theory. This highlights the need to be aware that strengthening personal responsibility and involvement might also inflict blame. Our findings imply that greater emphasis should be given to the systematic reflection and discussions among HLC professionals and their leaders about what user involvement implies in the local HLC context and in each user’s situation. Further research should investigate the users’ perspectives of involvement in the HLCs and look into how individual adjustment and user involvement are negotiated against resource use and standardization of services.

## Availability of data and material

The datasets used and/or analysed during the current study are available from the corresponding author on reasonable request.
